# Developing item banks to measure three important domains of health-related quality of life (HRQOL) in Singapore

**DOI:** 10.1186/s12955-019-1255-1

**Published:** 2020-01-02

**Authors:** Elenore Judy B. Uy, Lynn Yun Shan Xiao, Xiaohui Xin, Joanna Peck Tiang Yeo, Yong Hao Pua, Geok Ling Lee, Yu Heng Kwan, Edmund Pek Siang Teo, Janhavi Ajit Vaingankar, Mythily Subramaniam, Mei Fen Chan, Nisha Kumar, Alcey Li Chang Ang, Dianne Carrol Bautista, Yin Bun Cheung, Julian Thumboo

**Affiliations:** 1 Department of Rheumatology & Immunology, Singapore General Hospital, Singapore, Singapore; 2 Medicine Academic Clinical Programme, Singapore General Hospital, Singapore, Singapore; 3 Department of Physiotherapy, Singapore General Hospital, Singapore, Singapore; 4 Department of Social Work, Faculty of Arts and Social Sciences, National University of Singapore, Singapore, Singapore; 5 Program in Health Services and Systems Research, Duke-NUS Medical School, Singapore, Singapore; 6 Research Department, Institute of Mental Health, Singapore, Singapore; 7 Neuroscience and Mental Health, Lee Kong Chian School of Medicine, Singapore, Singapore; 8 Health Promotion Board, Singapore, Singapore; 9 Singapore Clinical Research Institute, Singapore, Singapore; 10 Centre for Quantitative Medicine, Duke-NUS Medical School, Singapore, Singapore; 11 Tampere Center for Child Health Research, University of Tampere and Tampere University Hospital, Tampere, Finland; 12 Office of Clinical, Academic & Faculty Affairs, Duke-NUS Medical School, Singapore, Singapore; 13 Yong Loo Lin School of Medicine, National University of Singapore, Singapore, Singapore

**Keywords:** Patient reported outcome measures, Quality of life, Singapore, Outcome assessment (health care), Survey and questionnaires, Adult, Psychometrics

## Abstract

**Objectives:**

To develop separate item banks for three health domains of health-related quality of life (HRQOL) ranked as important by Singaporeans – physical functioning, social relationships, and positive mindset.

**Methods:**

We adapted the Patient Reported Outcomes Measurement Information System Qualitative Item Review protocol, with input and endorsement from laymen and experts from various relevant fields. Items were generated from 3 sources: 1) thematic analysis of focus groups and in-depth interviews for framework (*n* = 134 participants) and item(*n* = 52 participants) development, 2) instruments identified from a literature search (PubMed) of studies that developed or validated a HRQOL instrument among adults in Singapore, 3) a priori identified instruments of particular relevance. Items from these three sources were “binned” and “winnowed” by two independent reviewers, blinded to the source of the items, who harmonized their selections to generate a list of candidate items (each item representing a subdomain). Panels with lay and expert representation, convened separately for each domain, reviewed the face and content validity of these candidate items and provided inputs for item revision. The revised items were further refined in cognitive interviews.

**Results:**

Items from our qualitative studies (51 physical functioning, 44 social relationships, and 38 positive mindset), the literature review (36 instruments from 161 citations), and three a priori identified instruments, underwent binning, winnowing, expert panel review, and cognitive interview. This resulted in 160 candidate items (61 physical functioning, 51 social relationships, and 48 positive mindset).

**Conclusions:**

We developed item banks for three important health domains in Singapore using inputs from potential end-users and the published literature. The next steps are to calibrate the item banks, develop computerized adaptive tests (CATs) using the calibrated items, and evaluate the validity of test scores when these item banks are administered adaptively.

## Introduction

Health has traditionally been measured by assessing the presence of disease, as seen in the use of mortality and morbidity statistics to compare health among various countries. However, with advances in medicine and public health, many diseases can be treated effectively, resulting in decreased morbidity and mortality. Thus, in addition to the traditional outcome measures, health has become defined as “a state of complete physical, mental and social well-being and not merely the absence of disease or infirmity” [1]. HRQOL instruments are empirical measures of this multidimensional and positive definition of health. They assess those areas of health patients experience and care about that are not addressed by conventional epidemiological measures such as morbidity and mortality.

Numerous instruments have been developed, validated, and used to measure health-related quality of life (HRQOL), these include instruments from the Patient-Reported Outcomes Measurement Information System (PROMIS), the World Health Organization Quality of Life (WHOQOL) group, the Short Form-36 (SF-36) of the Medical Outcomes Study, and the EuroQOL five-dimension questionnaire (EQ-5D). These generic instruments make possible the measurement of HRQOL across different disease conditions. Despite being more informative than traditional disease measures, existing HRQOL instruments are not without shortcomings. Valid HRQOL instruments must accurately reflect the experiences and priorities of the target population whose health it measures [2]. Although HRQOL instruments were intended to be used across different cultures, the fact remains that many of these instruments were developed and tested in the West, based on Western conceptions of health, and intended for use in Western cultural contexts. Although there has been significant effort to adapt these instruments to the Singapore context, several key issues remain. First, these instruments do not adequately account for the cultural differences between the West and Asia, and therefore do not accurately reflect the conceptualization, priorities, and experiences of health among people in Asia - this has been shown in studies done in Japan [3], China [4], Taiwan [5], and Singapore [6–8]. Second, although these instruments are viewed as sufficiently accurate to measure HRQOL on a population level, they generally do not measure HRQOL with enough precision to measure the health of individual patients over time [6, 7, 9, 10].

The reduced precision of inter-individual measurements are identified with the use of instruments which were developed using classic test theory [11]. These instruments are administered using a fixed set of items regardless of the respondent’s level of the latent trait being measured. This approach to health measurement results in instruments that are either highly precise but cover a small range of latent traits, or less precise but cover a larger range of latent traits, i.e. measurements which allow for depth or breadth of measurement, but not both [11]. The PROMIS initiative sought to overcome these limitations by using item response theory (IRT) and computerized adaptive testing (CAT) [11]. IRT allows scale developers the use of a larger selection of items to model the latent trait and identify the level of the latent trait each item measures. This allows for each of the items to be arranged on a scale, based on the level of latent construct each item measures [12]. CAT is a system for administering the test whereby the next item administered to a respondent is determined by his/her response to the previous administered item [12]. When used together with CAT, IRT makes possible the identification of a manageable number of items (a subset of the larger selection) likely to offer the most precision, to be administered to a given individual [12]. In order to achieve this, PROMIS investigators had to identify and develop items which cover the entire range of experience in the domains which the instrument was intended to measure [11].

Recognizing the need for an HRQOL instrument that adequately captures the conceptualization, priorities, and experience of health among Singaporeans, with sufficient precision at the population and individual level, we sought to develop domain-specific item banks through a multistage process: Stage 1: we used focus groups and in-depth interviews (*n* = 134 participants) to develop a health-domains framework which captures the Singapore population’s conceptualization of health [13]; Stage 2: we used a domain-ranking survey (*n* = 603 participants) to establish the importance-hierarchy of the 27 health domains in order to understand the health priorities of the Singapore population [14]. This domain-ranking survey led to the identification of the highly-ranked health domains [14].

In Stage 3, we aimed to develop item banks for three of these highly-ranked health domains: Physical Functioning, Social Relationships, and Positive Mindset. The process of developing these item banks is described in this paper.

The developed item banks were subsequently calibrated using IRT (Stage 4); results of the item-calibration survey for the item bank on Social Relationships, and Positive Mindset have been published [15, 16]. Once validated, the calibrated, domain-specific item banks can be developed into CATs to measure HRQOL.

## Methods

This study was reviewed and approved by the Singhealth institutional review board (CIRB Reference: 2014/916/A and 2016/2031) and has been conducted according to the principles expressed in the Declaration of Helsinki; written consent was obtained from all study participants.

**Fig. 1:**
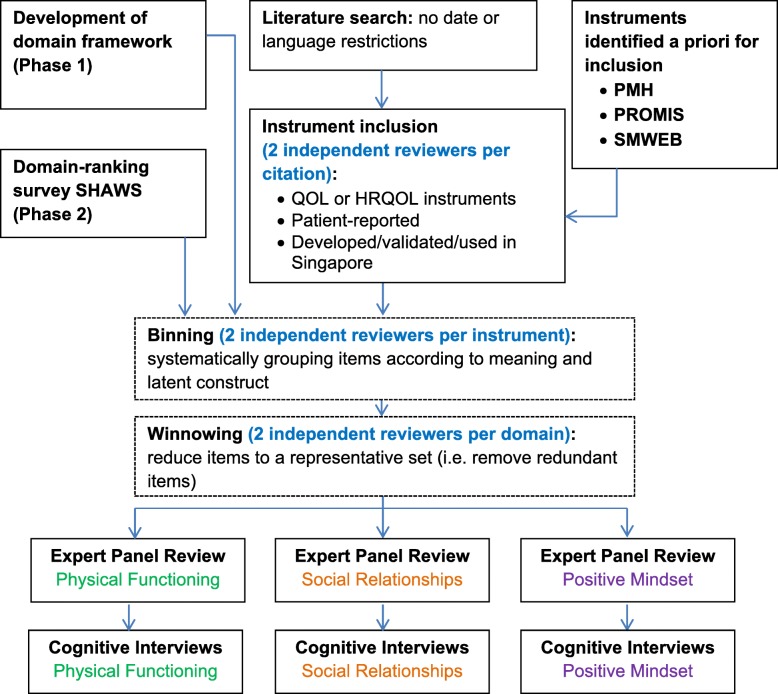
Overview of approach for developing domain-specific item banks. PMH-Positive Mental Health Instrument, PROMIS-Patient-Reported Outcomes Measurement Information System, SMWEB-Singapore Mental Wellbeing Scale

We generated items from 3 sources: 1) thematic analysis of our focus groups and in-depth interviews, 2) instruments identified from literature search, and 3) identified instruments of particular relevance. Items from these three sources were combined and underwent a stepwise, qualitative item review process using a modified version of the PROMIS Qualitative Item Review (QIR) protocol [11], which comprised of the following: item classification (“binning”) and selection (“winnowing”), item revision, item review by a panel of experts, cognitive interviews, and final revision (Fig. 1).

### Generating new items from qualitative studies

Participants of a previously completed domain-ranking survey (Stage 2) were community dwelling individuals, selected using a multi-stage sampling plan [14]. Singapore citizens or permanent residents, 21 years or older, of Chinese, Malay or Indian ethnicity, who spoke either English or Chinese (Mandarin), were eligible. Towards the end of the survey, participants were asked if they were willing to be contacted for future studies to further discuss their views on health; 46% were agreeable to be contacted. This subset of participants was purposively-sampled and invited to participate in the item-generation in-depth interviews.

We contacted potential participants via phone and set appointments to conduct the in-depth interview with those who were willing to participate. An experienced, female interviewer (YPTJ) with Master's-level training in sociology carried out the in-depth interviews face-to-face, in the participant’s home; this interviewer was not involved in the domain-ranking survey and had not previously interacted with the interview participants. All interviews were audio-recorded; the interviewer created field notes immediately after each interview.

Each participant was interviewed about each of the 3 shortlisted domains. The in-depth interview probes (Additional file 1) were designed to generate discussion about what characterizes each of the three shortlisted domains. For the domain Physical Functioning, each participant was asked to think of someone that they know who is able to function well physically and asked to describe what they observed about that person that made them think that they had good physical functioning. Similar probes were used for the domains of Social Relationships and Positive Mindset. Each audio-recorded interview was transcribed verbatim. Interviews conducted in Chinese were translated and transcribed in English.

YPTJ analyzed the transcripts using thematic analysis in Nvivo10 soon after each interview was completed. Identified themes were discussed by the study team (JT, EU, YX) on a weekly basis. We continued to recruit participants to the in-depth interviews until no new themes were generated, at which point the team reviewed the coverage of the identified themes and agreed that the in-depth interviews had reached saturation.

YPTJ then generated an initial set of items for each of the three shortlisted domains. These items were reviewed and refined by the study team sitting en bloc. In addition, a second study team member (EU), not involved in the in-depth interviews, reviewed the coded transcripts from our qualitative work to build a health-domains framework (Stage 1) and the item-generation in-depth interviews (Stage 3) to further refine the items. The revised set of items were reviewed and refined in a second study team meeting. This iterative process of transcript review and item refinement was carried out for all of the items generated from our qualitative study. Items were finalized after 3 iterations.

### Identifying existing instruments for inclusion

We searched PubMed using the following search terms: Quality of life or HRQOL, Patient Reported Outcomes or Questionnaire or Item Banks, Singapore, Adult, and the PubMed search filter for finding studies on measurement properties of measurement instruments developed by Terwee et al [17] The detailed electronic search strategy is listed in Additional file 2. We ran the search on November 3, 2015.

Two reviewers independently assessed each citation identified in the search for inclusion at the level of the study, then at the level of the instrument. Reviewers used a standard template to assess study inclusion, identify instruments used, document instrument characteristics, and assess instruments for inclusion. This template was designed and piloted for this study. In order to ensure that the selection was inclusive, studies and instruments identified for inclusion by at least one reviewer were included into the next step.

Studies were eligible for inclusion if they developed, validated, or used a quality of life instrument (generic or disease-specific), in an adult population in Singapore. Since the characteristics for study inclusion were not routinely reported in the title and abstract, reviewers assessed title, abstract, and full text together. For each included study, the name, version number, language, and Singapore cross-cultural adaptation status of all HRQOL instruments used were independently extracted by two reviewers.

Three instruments were a priori selected for inclusion: two locally developed instruments, the Positive Mental Health (PMH) instrument [18] and the Singapore Mental Wellbeing (SMWEB) Scale [19] whose developers collaborated in this study, and the relevant PROMIS domains [20] . All three instruments were included to enhance domain coverage in the resulting item banks.

The instrument names extracted from the previous step were consolidated to identify the instruments from which items for evaluation were extracted. In order to optimize the number of relevant items that reach the evaluation stage (below), when multiple versions of the same instrument were found across the included studies, we chose to include the most recent, locally adapted, and exhaustive (i.e. full instrument over the short form) version.

Two reviewers independently assessed each instrument for inclusion based on the following inclusion criteria: 1) measured a patient-reported outcome (proxy-reported measures were not included), and 2) had items that were relevant to at least one of the three shortlisted domains. To carry out this assessment, each reviewer obtained information about the instrument using the proprietary database for patient reported outcome measures, the Patient-Reported Outcome and Quality of Life Instruments Database (PROQOLID) [21], and internet searches. We also obtained copies of the shortlisted instruments either through sources available to the public, i.e. official websites or research publications, or by requesting copies from instrument developers or study investigators who used the instruments locally. This resulted in a final list of instruments from which we extracted items for evaluation. Each of the PROMIS domain instruments were likewise independently evaluated for inclusion by two reviewers (Additional file 3).

All items from the shortlisted instruments were extracted into a standard template that also captured instrument origin and stem question. This item library was used as the starting point for item evaluation.

### Item evaluation and revision

#### Item classification (binning)

As defined by the PROMIS Cooperative Group, “binning refers to a systematic process for grouping items according to meaning and specific latent construct”, the final goal of which was to have a bin with an exhaustive list of items from which a small number of items could be chosen to adequately represent the bin. This process facilitates recognition of redundant items and easy comparison to identify the most representative item within a given bin [11].

As the instruments which were eligible for inclusion all had an identified English version, binning was carried out using the English items. Binning was done in such a way that at least two independent reviewers evaluated any one item for possible inclusion. Each item was included in as many bins as a reviewer saw fit. In order to ensure that binning was exhaustive, an item identified for inclusion to a bin by at least one reviewer was included in that bin.

We undertook a two-stage process for binning. First-order binning was done at the level of the domain: reviewers evaluated each item for possible inclusion into Physical Functioning, Social Relationships, and/or Positive Mindset. Second-order binning was done at the level of the subdomain; within each first-order bin (domain), reviewers assessed each item for inclusion into a subdomain bin. Reviewers created bins based on emerging item categories, as they carried out the second-order binning. We did not set limits as to the number of bins that could be used. The final number of bins used for second-order binning was reached by consensus between reviewers.

#### Item selection (winnowing)

Upon completion of binning, a pair of reviewers independently assessed each of the bins and selected three items most representative of the bin. This process of reducing the large set of items down to a representative set of items is referred to as “winnowing” [11]. The process of winnowing was carried out separately for each domain and was guided by the domain definitions [14]; bins and items which fell outside the scope of these definitions were excluded.

After completing item selection independently, each pair of reviewers sat together with a third reviewer, not previously involved in the winnowing process, to identify which bins were not consistent with the study domain definitions and for removal, and which three of the items best represented the retained bins.

#### Item revision

The items which underwent binning and winnowing came from various instruments. They were created in varying styles, syntaxes, phrasing, and levels of literacy. To facilitate administration of the items as a coherent test, the study team standardized the format of the English items based on the following principles: 1) literacy level geared towards someone with standard GCE or ‘O’ level English (approximately 16 years old, with approximately 10 years of formal schooling), 2) used non-ambiguous, simple, and commonly-used words, 3) positively-worded and stated in the first person to facilitate understanding and relatability, 4) answerable by one of the PROMIS preferred response options to facilitate participant familiarity with a limited set of response options throughout the test (i.e. lessen cognitive burden) [11]. Item revision was carried out according to domain. After item revision was completed for each domain, we reviewed items across all domains in order to ensure parallel wording and statement construction within and across domains.

### Item-review (expert panel)

We convened an expert panel for each of the three domains. Each expert panel was comprised of five to seven content experts and lay representatives. Content experts were from various clinical (nurse, physiotherapist, clinical psychologist, physicians), research (health research, social research), and public health backgrounds. Two separate expert panel meetings were held for each of the domains.

The first meeting was to discuss domain definitions and the overall plan for creating item banks including the approach to developing items from our own qualitative studies and identifying instruments for inclusion, prior to undertaking the literature search.

The second meeting was for the expert panel to systematically assess the face and content validity of each of the shortlisted, reworded items and how successfully the group of items within a given domain achieved adequate coverage. Statement construction, wording, and appropriate PROMIS response options were considered at the level of the item. Test instructions, time frame, and domain coverage were considered at the level of the domain. We achieved consensus for proposed item revisions over two rounds of consultation (the first through face-to-face discussion during the panel meeting, the second through email correspondence.).

### Cognitive interviews

Participants for all cognitive interviews (details in Methods: Section F to H) were recruited from the outpatient clinics of the Singapore General Hospital – we included patients, caregivers, or members of the general public, regardless of health status. Participants were purposively sampled to ensure representation across gender, age group, and ethnicity within each cognitive interview iteration. Singaporeans and permanent residents, 21 years or older, who spoke English or Chinese were eligible to participate. Except for the qualifying language of interview, the process of recruiting participants and conducting the interview was similar for English-language and Chinese-language cognitive interviews.

Participants were interviewed face-to-face immediately after recruitment, in a relatively quiet area of the outpatient clinic where they were recruited. The cognitive interviews were carried out by a research staff trained in conducting cognitive interviews.

We hypothesized that test comprehension would be most influenced by age, gender, ethnicity, and education level. In Singapore, the level of education correlates with age, those in the younger age groups would have completed at least secondary-level education (i.e. 10 years of education). However, given the small sample size per cognitive interview iteration (4 participants), it was not possible to recruit participants across all these demographic strata.

Within each iteration of the English-language cognitive interviews, we sought to include at least one participant from each of the three ethnic groups (Chinese, Malay, Indian), a mix of both genders, and at least 1 participant from each of three age categories (21 to 34 years, 35 to 50 years, > 50 years).

We based our cognitive interview process on the PROMIS QIR protocol [11] and completed all English-language cognitive interviews and item revisions before proceeding to cross-culturally adapt the finalized English-language items in Chinese.

### English-language cognitive interviews to assess items and response options

We conducted cognitive interviews to elicit participant feedback and input on the comprehensibility, wording, and relevance of each item. We also solicited participant feedback on the test instructions, time frame, and overall level of difficulty of the test.

To minimize respondent fatigue, we solicited input from each participant for at most 42 items spanning up to 3 domains. We allowed participants to self-complete the pen-and-paper questionnaire (instructions, time frame, items, and response options) using the version endorsed by the expert panels. Immediately after the participant completed the test, a trained interviewer systematically reviewed the test with the participant and elicited how the participant understood the test and arrived at a response for each of the items. This method of retrospectively probing about the participant’s thought process after he/she had already completed the test is consistent with the intended use of the calibrated item banks as a self-administered test [22]. Although this method of probing is subject to limitations of recall, it minimizes the risk that the interviewer’s questions may bias the way participants respond to the test [22]. Also, by probing immediately after the questionnaire was completed, there was a higher chance that participants would be able to recall the thought process that underpinned their responses. In instances where the participant did not understand an item, the trained interviewer explained the intent of the item and inquired on how the participant would reword the item given the stated intent.

Items were revised iteratively based on inputs from the cognitive interviews. Each iteration was based on the input from three to four cognitive interview participants. For items that underwent substantial revision, we ensured that the revised item was evaluated and found satisfactory in at least two rounds of iteration before finalizing the revision.

Each response set in the PROMIS list of preferred response options is comprised of five descriptors along a Likert-type response scale, e.g. for Frequency, the descriptors were “Never”, “Rarely”, “Sometimes”, “Often”, “Always” [11]. Part of the cognitive interviews was focused on assessing how participants selected a response for each item and how they ascribed value to the descriptors within a given response set. The latter was assessed by a card sort activity [23] [24] which was carried out at the start of the cognitive interview, immediately after the participant has completed the self-administered test. We prepared five printed cards, each card bearing one of the five descriptors, which were shuffled after each use. During the card sort, participants were asked to order the cards in ascending order. After the participant had ordered the cards, the interviewer verbally confirmed the suggested order with the participant and probed on the reasons behind the suggested order.

### Developing the English-language response scale for use in Singapore

During the above cognitive interviews, it became apparent that despite having seen the PROMIS ordering of these descriptors in the course of completing the self-administered test, the participants’ perception of how these descriptors should be ordered did not match those in PROMIS. In addition, we noted that participants selected a response either 1) by ascribing value based solely on the wording of each descriptor or 2) by ascribing value to a descriptor, based on its position relative to other descriptors within a given response set (details in the Results section). In order to elicit meaningful responses from both types of participants, we felt it was necessary that each descriptor within a response set was worded in a way that it was consistently valued across both types of potential end users.

We adapted the WHOQOL standardized method for developing a response scale across different languages and cultural contexts to develop separate sets of descriptors for “Capability”, “Frequency”, and “Intensity” [23] [24]. First, we generated a list of possible anchors (i.e. descriptors for the “floor” and “ceiling” of each set of response options; for “Frequency”, these descriptors would be “Never” and “Always”, respectively) and intermediate descriptors based on the WHOQOL list of anchors and descriptors [23] and the PROMIS preferred response options [11]. We supplemented this list by using online dictionaries and thesauruses to search for synonyms of the initial set of descriptors. For each response set, cognitive interview participants were asked to select which of the “floor” and “ceiling” descriptors had the lowest and highest value respectively. The most commonly selected floor and ceiling descriptors were used as anchors in the exercise to select the intermediate descriptors for the response options for local use.

We recruited 30 participants in order to formally measure the magnitude of the candidate intermediate descriptors. To ensure representation across demographics, we instituted quotas for gender, ethnicity, and education level. All participants gave input on four response sets: two sets for “Capability”, and one set each for “Frequency” and “Intensity”.

The activity to measure the magnitude of the intermediate descriptors used 14 to 18 candidate intermediate descriptors for each response set. Each descriptor was printed on a piece of A4-sized paper alongside an unmarked 100-mm line bounded by a “floor” and “ceiling” descriptor on either end. Participants were instructed to mark with a pen, a point between the two anchors that corresponds to the value of the descriptor.

A single trained interviewer administered the activity to all study participants. The interviewer administered a sample activity before proceeding to the full activity. Prior to the start of each response set, the interviewer introduced the concept being measured (i.e. capability, frequency, intensity), ensuring that the participant understood the concept before proceeding to the activity. Intermediate descriptors were administered according to response set; a short cognitive interview was carried out immediately after each completed response set.

Each participant’s rating for an intermediate descriptor was measured as the distance (in millimeters) from the lower end of the unmarked line (corresponding to the position of the “floor descriptor”) to the point on the line which was marked by the participant. For each response set, the mean and standard deviation for each of the intermediate descriptors was calculated. Similar to the WHO standard method, we selected three intermediate descriptors per response set, one descriptor each for each of the following ranges: 20–30 mm; 45–55 mm; 70–80 mm. If more than one descriptor fell in a given range, the one with the lowest standard deviation was selected [23].

### Chinese cross-cultural adaptation of items and response options

We completed all English test cognitive interviews and revisions before proceeding to cross-culturally adapt the finalized items in Chinese following the recommended guidelines [25]. Two sets of translators independently carried out forward translation (English to Chinese) and back translation (Chinese to English). All translators, along with study team members, subsequently discussed the wording of the items and response options to resolve any differences between the English and Chinese versions. The cross-culturally adapted Chinese-language items were then tested in cognitive interviews with Chinese-language participants.

Within each iteration of the Chinese-language cognitive interviews (3 to 4 participants per iteration), we sought to include a mix of both genders, and at least 1 participant from each of three age categories (21 to 34 years, 35 to 50 years, > 50 years).

For the response scales, the Chinese-language descriptors were tested using card sorts and cognitive interviews. Each participant completed card sorts for three response sets: “Intensity”, “Frequency”, and “Capability 1”. We planned to carry out a formal process of developing the Chinese-language response scale using the WHOQOL standardized method should the card sort reveal that the descriptors were not consistently valued by participants.

### Intellectual property and seeking permission from instrument developers

Similar to PROMIS, the intent is to make the instrument arising from this study freely available to clinicians and researchers for non-commercial use. We therefore formally reviewed the finalized items to determine if the developers of the source instruments have reasonable claim of intellectual property for the items which emerged after several rounds of revisions based on participant, expert panel, and study team inputs.

## Results

**Fig. 2:**
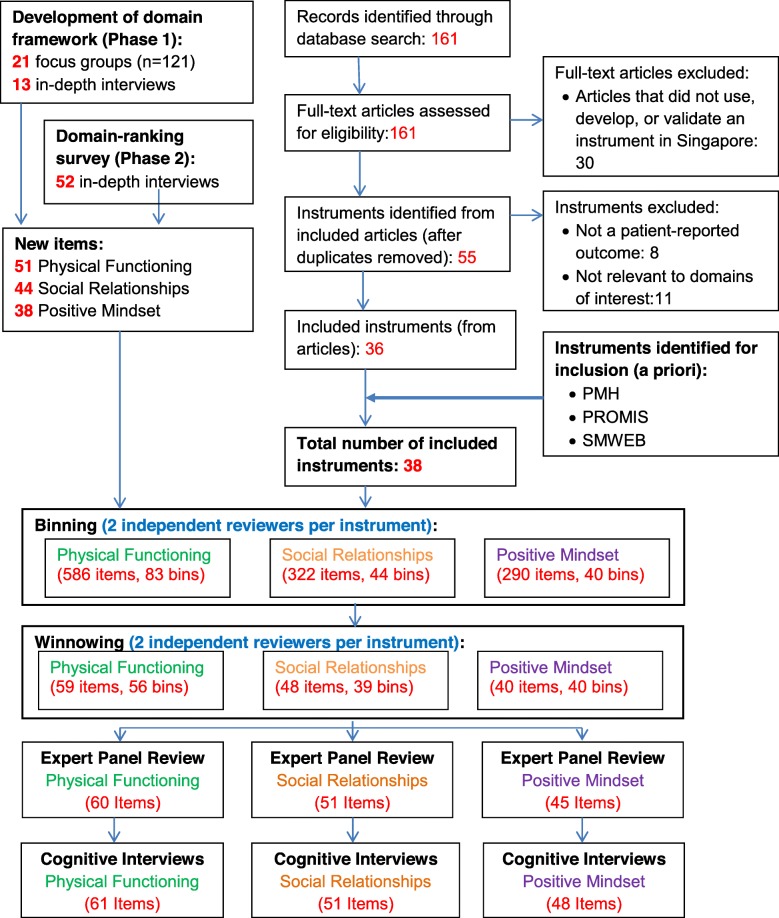
Results of multistep approach for developing domain-specific item banks. PMH-Positive Mental Health Instrument, PROMIS-Patient-Reported Outcomes Measurement Information System, SMWEB-Singapore Mental Wellbeing Scale

The results at the end of each step of our multistep process to developing domain-specific item banks are summarized in Fig. 2.

### Generating new items from qualitative studies

We conducted in-depth interviews with 52 community-based individuals from September to November 2016. Interviews were conducted in English or Mandarin with the exception of bilingual speakers, among whom some interviews were conducted using both languages. Most of the interviews were conducted in the participant’s home; in some instances, participants had to end the interview prematurely due to pressing domestic concerns. The average duration of the interviews was 21.4 min (Standard Deviation (SD): 8.7 min, Range: 6 to 50 min); 90% of interviews lasted more than 10 min.

The demographic profile of the in-depth interview participants is summarized in Table 1. The mean age of participants was 45.3 years (SD: 15.7 years, Range: 24 to 79 years).

**Table 1:** Demographic profile of in-depth interview participants

		Frequency	Percentage
Gender	Male	22	42
	Female	30	58
Age Category	21 to 34 years	16	31
	35 to 49 years	17	33
	50 years and older	19	36
Ethnicity	Chinese	20	38
	Malay	17	33
	Indian	15	29
Language spoken	English only	17	33
	Chinese only	3	6
	Bilingual (English and Chinese)	10	19
	Bilingual (English and Malay)	14	27
	Bilingual (English and Tamil)	8	15
Years of Education	0 to 6 years	5	10
	7 to 12 years	24	46
	≥ 13 years	23	44
Marital status	Single	9	17
	Married	39	75
	Divorced	1	2
	Widowed	2	4

We generated 133 items from our qualitative studies: 51 Physical Functioning, 44 Social Relationships, and 38 Positive Mindset.

### Identifying existing instruments for inclusion

The implemented PubMed search identified 161 citations. Review of these citations identified 55 unique instruments which were developed, validated, or used in an adult cohort in Singapore; 36 of these instruments were patient-reported, with items which were relevant to the shortlisted domains. A flow diagram of the yield at each stage of the literature review is included in Fig. 2; the 19 instruments excluded after evaluation are listed in Additional file 4. The PMH instrument, one of the 3 instruments identified for inclusion a priori, was also identified from the literature review. These 36 instruments, together with the SMWEB and PROMIS instruments, comprised the 38 instruments (Table 2) which were included in the item library. Within PROMIS, 18 domain instruments were included; the items in these instruments were likewise included in the item library.

**Table 2:** List of instruments included in the item library

Generic Instruments	Disease-Specific Instruments
• DETERMINE Your Nutritional Health Checklist [26] • EuroQoL 5-Dimension (EQ-5D) Instrument [27] • Medical Outcomes Study Family and Marital Functioning Measures [28] • 12-item General Health Questionnaire (GHQ-12) [29] • Health Utilities Index (HUI) [30] • Lequesne Algofunctional Index^b^ [31] • Life Satisfaction Index (LSI) [32] • Oxford Knee Score (OKS) [33] • Physical Self-Maintenance Scale (PSMS) [34] • Positive Mental Health (PMH) Instrument [18] • Satisfaction with Life Scale (SWLS) [35] • 36-Item Short Form Health Survey (SF-36) [36] • Singapore Successful Aging Questionnaire^b^ [37] • Singapore Mental Wellbeing (SMWEB) Scale [19] • Patient-Reported Outcomes Measurement Information System (PROMIS) Instrument^a^ [11]	• Beck Anxiety Inventory (BAI) [38] • Caregiver Quality of Life Index-Cancer (CQOLC) [39] • Center for Epidemiologic Studies Depression Scale (CES-D) [40] • NCCN Distress Thermometer and Problem List [41] • European Organisation for Research and Treatment of Cancer Core Quality of Life Questionnaire (EORTC QLQ-C30) [42] • Functional Assessment of Cancer Therapy - Breast (FACT-B) [43] • Functional Assessment of Cancer Therapy - Gastric (FACT-Ga) [44] • Functional Assessment of Cancer Therapy - General (FACT-G) [45] • Functional Living Index-Cancer (FLIC)^b^ [46, 47] • 36-item Glaucoma Quality of Life (Glau-QoL-36) Questionnaire [48] • Hepatitis Quality of Life Questionnaire (HQLQ)^b^ [49] • 30-Item Hemifacial Spasm Questionnaire (HFS-30) [50] • Hospital Anxiety and Depression Scale (HADS) [51] • Kidney Disease Quality of Life Short Form™ (KDQOL-SF™) [52] • Knee injury and Osteoarthritis Outcome Score (KOOS)^bc^ [7] • Medical Outcomes Study HIV Health Survey (MOS-HIV) [53] • Parkinson’s Disease Questionnaire (PDQ-39) [54] • Quality of Life–Alzheimer’s Disease (QOL-AD) Scale [55] • Rheumatology Attitudes Index (RAI) [56] • Schizophrenia Quality of Life Scale (SQLS) [57] • Scleroderma Health Assessment Questionnaire (SHAQ)^b^ [58] • State-Trait Anxiety Inventory (STAI) [59] • Systemic Sclerosis Quality of Life Scale (SSc-QoL) [60]

^a^Included PROMIS domain instruments listed in Additional file 3

^b^Instruments which are locally-adapted versions of HRQOL instruments originally developed outside of Singapore

^c^ The KOOS includes the Western Ontario and McMaster Universities (WOMAC) Osteoarthritis in its complete and original format [61] [62]. For purposes of this study, the WOMAC was considered a subset of the KOOS

Instruments identified for inclusion were in English, Chinese, Tamil, or Malay. Majority of the instruments were in English, or English and Chinese.

### Item evaluation and revision

Reviewers who independently binned items from the library created 167 bins: 83 bins for Physical Functioning, 44 bins for Social Relationships, and 40 bins for Positive Mindset. On average, each of these bins had 10 items (SD: 10.0, Range: 1 to 55) for Physical Functioning, 10 items (SD: 10.2, Range: 1 to 45) for Social Relationships and 9 items (SD: 7.7, Range 1 to 36) for Positive Mindset. At the stage of winnowing, reviewers opted to 1) remove 37 bins which were not consistent with the study’s domain definitions: 27 Physical Functioning, 5 Social Relationships, and 5 Positive Mindset respectively; 2) add 5 bins for positive mindset (These bins were initially identified as sub-themes of other bins; on further discussion, these were found to be conceptually distinct and of sufficient importance to be a stand-alone bins). The reviewers identified representative items for all 135 bins: 56 bins for Physical Functioning, 39 bins for Social Relationships, and 40 bins for Positive Mindset. All the items which were shortlisted by reviewers at the winnowing stage underwent item revision prior to review by domain-specific expert panels.

### Item-review (expert panel)

Expert panel members reviewed all of the items and provided input on the wording and appropriate response option for each item. Fifteen items were omitted due to redundancy (Physical Functioning: 1, Social Relationships: 13, Positive Mindset: 1). twenty-four items were added to improve domain coverage (Physical Functioning: 2, Social Relationships: 16, Positive Mindset: 6). Following the expert panel review, we had 156 items in our pool: (Physical Functioning: 60, Social Relationships: 51, Positive Mindset: 45). In four instances, where the expert panel was unable to decide between two or more competing revisions for the same item, the expert panel suggested that these items be tested side by side during the cognitive interviews in order to select the version of the item which was most suited for use in the Singapore population. As such, 160 items (Physical Functioning: 61, Social Relationships: 51, Positive Mindset: 48) were assessed during the cognitive interviews (Additional file 5).

For all three of the shortlisted domains, expert panels endorsed the test instructions adapted from PROMIS. The time frame endorsed for Physical Functioning was one week; the time frame for Social Relationships and Positive Mindset was fourweeks. The expert panel’s choice for time frame was guided by considerations related to the period of recall and the period within which an intervention is expected to alter the measured outcome [2]. Although the appropriate response option was selected at the level of each item, the PROMIS response options for “Capability” and “Frequency” were consistently selected for items in the domain of Physical Functioning; the response options for “Frequency” and “Intensity” were consistently selected for items in the domains of Social Relationships and Positive Mindset.

### Cognitive interviews

All cognitive interviews (Section F to H) were carried out from March to July 2016. Participant characteristics are reported in their respective sections.

### English-language cognitive interviews to assess items and response options

We recruited 45 participants for the English cognitive interviews: mean age 45.1 years old (SD: 14.3, Range: 21 to 70), 44.4% Female, 44.4% Chinese, 22.2% Malay, 33.3% Indian. We finalized the wording for 160 items (Physical Functioning: 61, Social Relationships: 51, Positive Mindset: 48) after 7 iterations of interviews.

The first 21 participants recruited to the English cognitive interviews participated in the card sort activity. All participants in the “Capability 1” card sorts (*n* = 8) arranged the descriptors in the hypothesized order. However, 58.8% of the “Intensity” (*n* = 17) and 18.8% of the “Frequency” (*n* = 16) card sort participants arranged the descriptors differently from the hypothesized order. We noted that participants used the proffered response options in at least two different ways. The majority of participants accepted the ordering of the descriptors as they were provided. These participants took cues from the descriptors that anchored the response set (e.g. for Frequency, the anchors were “Never” and “Always”), assessed where along the continuum between the two anchors their response would fall, and selected the descriptor that corresponded to that distance. A smaller number of participants evaluated each of the response descriptors alongside the item and selected the response descriptor that “felt” most appropriate (e.g. a participant repeatedly read out the item followed by each one of the descriptors, in order to evaluate which descriptor “felt” right).

### Developing the English-language response scale for local use

The following anchors were used for the activity (“Floor” descriptor, “Ceiling” descriptor): Capability 1: Without any difficulty, Unable to do; Capability 2: Unable to do, Completely able to do; Frequency: Never, Always; Intensity: Not at all, Extremely. We chose to test two Capability response sets, Capability 1 which expressed capability as extent of difficulty to do (negatively-worded), and Capability 2 which expressed extent of the ability to do (positively-worded). Capability 1 is based on the PROMIS preferred response options; Capability 2 was suggested by some of the cognitive interview participants.

We recruited 30 participants to develop the English response scale for local use: mean age: 47.2 years old (SD: 16.0, Range: 23 to 78), 53.3% Female, 33.3% Chinese, 33.3% Malay, 33.3% Indian, 13.3% with less than secondary level education. Based on the results (Additional file 6: Table S1) and the intermediate descriptor values recommended in the WHO standard method document, the study team decided to proceed with the Capability 1 set of descriptors. Table 3 summarizes the means and standard deviations of the shortlisted intermediate descriptors.

**Table 3:** Means and standard deviations for the shortlisted intermediate descriptors using a 100 mm Visual Analogue Scale

	25 mm descriptor	Mean (SD)	50 mm descriptor	Mean SD	75 mm descriptor	Mean (SD)
Intensity Anchors: 0 mm: *Not at all* 100 mm: *Extremely*	Mildly	27.83 (18.86)	Moderately	46.67 (10.49)	Quite a lot	78.40 (15.27)
Frequency Anchors: 0 mm: *Never* 100 mm: *Always*	Seldom	20.37 (11.48)	Sometimes	39.70 (17.62)	Usually	77.67 (14.32)
Capability 1 Anchors: 0 mm: *Unable to do* 100 mm: *Without any difficulty*	With much difficulty	17.17 (14.06)	With moderate difficulty	52.83 (16.59)	With minor difficulty	80.03 (18.88)

### Chinese cross-cultural adaptation of items and response options

We recruited 18 participants for the Chinese cognitive interviews: mean age 45.3 years old (SD: 12.9, Range: 22 to 64), 10 Female (55.6%). We finalized the wording for 160 items (Physical Functioning: 61, Social Relationships: 51, Positive Mindset: 48) after 3 iterations.

We recruited 6 participants to the card sorts using the Chinese-language response scale: mean age 47.5 years old (SD: 15.4, Range: 22 to 64), 66.7% Female, 33.3% with less than secondary level education. For the response sets for “Intensity”, “Frequency”, and “Capability 1”, 83.3, 66.6, and 100% respectively, were able to order the descriptors in the expected order.

### Intellectual property and seeking permission from instrument developers

We identified 53 items from 9 developers for which we sought written permission to modify and use as part of our item banks (Additional file 7: Table S2). The developers of 2 instruments declined to give permission for 3 items. To maintain representation of these items’ respective subdomains, they were replaced with other shortlisted items from the same bin: two from our own qualitative work: “I am a cheerful person” and “I am bedridden”, and one from the PMH: “When I feel stressed I try to see the humorous side of the situation”.

## Discussion

This paper summarises the process of developing item banks for three health domains: Physical Functioning, Social Relationships, and Positive Mindset. We used a multistep method in order to capture the conceptualization, priorities, and experience of these health domains among Singaporeans. To our knowledge, few HRQOL item banks were originally developed in Asia [63–65].

Our method of starting with a combined pool of items from our own qualitative work and existing instruments, and then subjecting all items to a qualitative item review process, allowed us to systematically develop our own items and scrutinize them alongside previously existing, validated items. The advantage of this approach is that it allowed us to generate items that captured the Singapore population’s experience and conceptualization of the domains and subdomains, while capitalizing on the face and content validity of existing validated items, and the domain coverage of existing instruments, to better ensure that the set of items included in the final item banks are able to measure the intended construct. In addition, working with the developers of the Positive Mental Health (PMH) Instrument and the Satisfaction with Life Scale (SWLS), researchers from the Institute of Mental Health and the Health Promotion Board respectively, made it possible to build the item banks on Social Relationships and Positive Mindset on existing static instruments which were developed in Singapore.

We initially used the PROMIS list of preferred response options to elicit participant response to each of the English-language items. Each response option was composed of five descriptors along a Likert-type response scale. In the course of our cognitive interviews, it became apparent that participants did not fully comprehend the meaning of the descriptors. On formal testing, less than 60% of participants could order the descriptors for “Intensity” in the order proposed by PROMIS. Similarly, less than 20% of participants could order the descriptors for “Frequency” in the proposed order. We therefore developed separate sets of descriptors for “Capability”, “Frequency”, and “Intensity” response options using the WHOQOL standardized method for developing a response scale across different languages and cultural contexts. The resulting set of revised response options was then translated into Chinese. More than 60% of participants were able to arrange the descriptors of the revised response options in the proposed order.

The next step towards using these domain-specific items banks in CATs is to calibrate them using IRT and evaluate the validity of test scores when these item banks are administered adaptively. To date, item-calibration of the domain specific item banks have been completed [15, 16]. Once fully developed, it is hoped that the resulting domain-specific instruments would make possible more precise measurements of HRQOL at the population and individual level, most especially in Singapore and Asia. Such an instrument would allow clinicians to measure and compare a patient’s HRQOL from consult to consult. Alongside other clinical measures (e.g. pain scores, functional class, etc.), these measurements would enable clinicians to evaluate the effectiveness of implemented treatment interventions and make timely modifications when necessary.

We acknowledge a number of study limitations. Due to limited resources, the systematic review to identify instruments that had been developed or validated among adults in Singapore was carried out on a single database (PubMed), using a precise search strategy, in a single language of publication (English). This approach opens the possibility that more obscure instruments reported in non-English language publications may have been missed by our search. Although the search was carried out in 2015, we did not deem it appropriate to update the search given the following reasons: 1) HRQOL instruments tend to remain unchanged and relevant for extended periods; many of the most highly accepted, validated instruments have been in their current form for more than a decade, 2) the manuscript describes part of a multi-stage process, of which the systematic review was an intervening step; presenting the search yield as it was carried out in 2015 allows for a clear understanding of the temporality and provenance of the items that were ultimately included in the item banks. If information about a new instrument was published for the first time after the search, it would not have been included in our study’s pool of instruments. Nevertheless, the search yielded 161 citations which identified 55 unique instruments, 36 of which were found to be relevant to the item banks being developed in this study. This, together with the a priori decision to include items from PROMIS, the SWLS, and the PMH instrument, and the substantial number of subdomains identified during binning and winnowing improves our confidence that adequate domain coverage was accomplished with the resulting number of candidate items. Also, although the literature search was carried out in English, we did not impose any language restriction on the instruments identified from the citations. The search primarily yielded English and Chinese language instruments. This is consistent with the common languages used in Singapore [66] and aligns with the study’s objective to develop item banks in English and Chinese.

## Conclusion

A multistep approach that combines inputs from qualitative studies and published literature allows for the systematic development of items to measure health domains. Using this method, we developed item banks for three important health domains in Singapore using inputs from potential end-users and the published literature. The next steps are to calibrate the item banks, develop CATs using the calibrated items, and evaluate the validity of test scores when these item banks are administered adaptively.

## Supplementary information

**Additional file 1.** In-depth interview probes.

**Additional file 2.** PubMed electronic search strategy for quality of life instruments validated in a Singapore, adult population.

**Additional file 3.** Patient Reported Outcomes Measurement Instrument System (PROMIS) Domain Instruments included in the item library.

**Additional file 4.** Instruments excluded from the item library.

**Additional file 5.** Domain-specific item banks.

**Additional file 6 Table S1.** Means and standard deviations for all tested intermediate descriptors.

**Additional file 7 TableS2.** Items for which instrument developer permission was sought.

## Data Availability

Data can be requested from the corresponding author.

## Abbreviations

BAI: Beck Anxiety Inventory
CAT: Computerised adaptive test
CES-D: Center for Epidemiologic Studies Depression Scale
CQOLC: Caregiver Quality of Life Index-Cancer
EORTC-QLQ-C30: European Organisation for Research and Treatment of Cancer Core Quality of Life Questionnaire
EQ-5D: EuroQoL 5-Dimension
FACT-B: Functional Assessment of Cancer Therapy - Breast
FACT-G: Functional Assessment of Cancer Therapy - General
FACT-Ga: Functional Assessment of Cancer Therapy - Gastric
FLIC: Functional Living Index-Cancer
GCE: General Certificate of Education
GHQ-12: 12-item General Health Questionnaire
Glau-QOL-36: 36-item Glaucoma Quality of Life
HADS: Hospital Anxiety and Depression Scale
HFS-30: 30-Item Hemifacial Spasm Questionnaire
HQLQ: Hepatitis Quality of Life Questionnaire
HRQOL: Health-Related Quality of Life
HUI: Health Utilities Index
IRT: Item Response Test
KDQOL-SF™: Kidney Disease Quality of Life Short Form™
KOOS: Knee injury and Osteoarthritis Outcome Score
LSI: Life Satisfaction Index
MOS-HIV: Medical Outcomes Study HIV Health Survey
OKS: Oxford Knee Score
PDQ-39: Parkinson’s Disease Questionnaire
PMH: Positive Mental Health
PROMIS: Patient-Reported Outcomes Measurement Information System
PROQOLID: Patient-Reported Outcome and Quality of Life Instruments Database
PSMS: Physical Self-Maintenance Scale
QIR: Qualitative Item Review
QoL-AD: Quality of Life–Alzheimer’s
RAI: Rheumatology Attitudes Index
SF-36: 36-Item Short Form Health Survey
SHAQ: Scleroderma Health Assessment Questionnaire
SMWEB: Singapore Mental Wellbeing
SQLS: Schizophrenia Quality of Life Scale
SSc-QoL: Systemic Sclerosis Quality of Life Scale
STAI: State-Trait Anxiety Inventory
SWLS: Satisfaction with Life Scale
WHOQOL: World Heatlh Organisation Quality of Life
WOMAC: Western Ontario and McMaster Universities
