# Supplementary material for: Developing item banks to measure three important domains of health-related quality of life (HRQOL) in Singapore
Source: Health Qual Life Outcomes. 2020 Jan 2;18:2. doi: 10.1186/s12955-019-1255-1 (PMC6941315; doi:10.1186/s12955-019-1255-1)
Supplement: Supplementary file 1 — Additional file 1. In-depth interview probes. [file 12955_2019_1255_MOESM1_ESM.docx]

**SUPPORTING INFORMATION**

# **Additional file 1. In-depth interview probes**

**Physical functioning**

Think of someone you know who is able to function well physically.

- What made you say that this person had good physical functioning?

OR

- What did you observe about them that made you think that they had good physical functioning?

Think of someone you know who is unable to function well physically.

- What made you say that this person has not functioned well physically?

OR

- What did you observe about them that made you think that they did not function well physically?

OR

- Could you give me examples that show that this person has not functioned well physically?

**Social Relationships**

Think of someone you know who has good relationship with others.

- What made you say that this person had a good relationship with others?

OR

- What did you observe about them that made you think that they had a good relationship with others?

Think of someone you know who has a difficult relationship with others.

- What made you say that they had a difficult relationship with others?

OR

- What did you observe about them that made you think that they had a difficult relationship with others?

OR

- Could you give me examples that show that this person has had a difficult relationship with others?

**Positive Mindset**

Think of someone you know who has a positive mindset.

- What made you say that this person has a positive mindset?

OR

- What did you observe about them that made you think that they had a positive mindset?

Think of someone you know who does not have a positive mindset.

- What made you say that this person does not have a positive mindset?

OR

- What did you observe about them that made you think that they did not have a positive mindset?

OR

- Could you give me examples that show that this person does not have a positive mindset?
