# Supplementary material for: Developing item banks to measure three important domains of health-related quality of life (HRQOL) in Singapore
Source: Health Qual Life Outcomes. 2020 Jan 2;18:2. doi: 10.1186/s12955-019-1255-1 (PMC6941315; doi:10.1186/s12955-019-1255-1)
Supplement: Supplementary file 2 — Additional file 2. PubMed electronic search strategy for quality of life instruments validated in a Singapore, adult population. [file 12955_2019_1255_MOESM2_ESM.docx]

# **Additional file 2. Pubmed electronic search strategy for quality of life instruments validated in a Singapore, adult population**

**Search ((((((((((("Quality of Life"[Mesh]) OR quality of life[tiab])) OR "health-related quality of life")) AND (((((((("Patient Outcome Assessment"[Mesh]) OR ((patient-reported outcome*) OR (((patient-reported outcome) OR patient-reported outcome measure) OR patient-reported outcome assessment)))) OR patient-administ*) OR self-administ*) OR self-complet*)) OR (((("Questionnaires"[Mesh]) OR questionnaire*) OR item*) OR item bank*))) AND ((Singapore[MeSH]) OR Singapore)) AND Adult[MeSH]))) AND (instrumentation[sh] OR Validation Studies[pt] OR "reproducibility of results"[MeSH Terms] OR reproducib*[tiab] OR "psychometrics"[MeSH] OR psychometr*[tiab] OR clinimetr*[tiab] OR clinometr*[tiab] OR "observer variation"[MeSH] OR observer variation[tiab] OR "discriminant analysis"[MeSH] OR reliab*[tiab] OR valid*[tiab] OR coefficient[tiab] OR "internal consistency"[tiab] OR (cronbach*[tiab] AND (alpha[tiab] OR alphas[tiab])) OR "item correlation"[tiab] OR "item correlations"[tiab] OR "item selection"[tiab] OR "item selections"[tiab] OR "item reduction"[tiab] OR "item reductions"[tiab] OR agreement[tw] OR precision[tw] OR imprecision[tw] OR "precise values"[tw] OR test-retest[tiab] OR (test[tiab] AND retest[tiab]) OR (reliab*[tiab] AND (test[tiab] OR retest[tiab])) OR stability[tiab] OR interrater[tiab] OR inter-rater[tiab] OR intrarater[tiab] OR intra-rater[tiab] OR intertester[tiab] OR inter-tester[tiab] OR intratester[tiab] OR intra-tester[tiab] OR interobserver[tiab] OR inter-observer[tiab] OR intraobserver[tiab] OR intra-observer[tiab] OR intertechnician[tiab] OR inter-technician[tiab] OR intratechnician[tiab] OR intra-technician[tiab] OR interexaminer[tiab] OR inter-examiner[tiab] OR intraexaminer[tiab] OR intra-examiner[tiab] OR interassay[tiab] OR inter-assay[tiab] OR intraassay[tiab] OR intra-assay[tiab] OR interindividual[tiab] OR inter-individual[tiab] OR intraindividual[tiab] OR intra-individual[tiab] OR interparticipant[tiab] OR inter-participant[tiab] OR intraparticipant[tiab] OR intra-participant[tiab] OR kappa[tiab] OR kappa's[tiab] OR kappas[tiab] OR "coefficient of variation"[tiab] OR repeatab*[tw] OR ((replicab*[tw] OR repeated[tw]) AND (measure[tw] OR measures[tw] OR findings[tw] OR result[tw] OR results[tw] OR test[tw] OR tests[tw])) OR generaliza*[tiab] OR generalisa*[tiab] OR concordance[tiab] OR (intraclass[tiab] AND correlation*[tiab]) OR discriminative[tiab] OR "known group"[tiab] OR "factor analysis"[tiab] OR "factor analyses"[tiab] OR "factor structure"[tiab] OR "factor structures"[tiab] OR dimensionality[tiab] OR subscale*[tiab] OR "multitrait scaling analysis"[tiab] OR "multitrait scaling analyses"[tiab] OR "item discriminant"[tiab] OR "interscale correlation"[tiab] OR "interscale correlations"[tiab] OR ((error[tiab] OR errors[tiab]) AND (measure*[tiab] OR correlat*[tiab] OR evaluat*[tiab] OR accuracy[tiab] OR accurate[tiab] OR precision[tiab] OR mean[tiab])) OR "individual variability"[tiab] OR "interval variability"[tiab] OR "rate variability"[tiab] OR "variability analysis"[tiab] OR (uncertainty[tiab] AND (measurement[tiab] OR measuring[tiab])) OR "standard error of measurement"[tiab] OR sensitiv*[tiab] OR responsive*[tiab] OR (limit[tiab] AND detection[tiab]) OR "minimal detectable concentration"[tiab] OR interpretab*[tiab] OR (small*[tiab] AND (real[tiab] OR detectable[tiab]) AND (change[tiab] OR difference[tiab])) OR "meaningful change"[tiab] OR "minimal important change"[tiab] OR "minimal important difference"[tiab] OR "minimally important change"[tiab] OR "minimally important difference"[tiab] OR "minimal detectable change"[tiab] OR "minimal detectable difference"[tiab] OR "minimally detectable change"[tiab] OR "minimally detectable difference"[tiab] OR "minimal real change"[tiab] OR "minimal real difference"[tiab] OR "minimally real change"[tiab] OR "minimally real difference"[tiab] OR "ceiling effect"[tiab] OR "floor effect"[tiab] OR "Item response model"[tiab] OR IRT[tiab] OR Rasch[tiab] OR "Differential item functioning"[tiab] OR DIF[tiab] OR "computer adaptive testing"[tiab] OR "item bank"[tiab] OR "cross-cultural equivalence"[tiab])) NOT (("addresses"[Publication Type] OR "biography"[Publication Type] OR "case reports"[Publication Type] OR "comment"[Publication Type] OR "directory"[Publication Type] OR "editorial"[Publication Type] OR "festschrift"[Publication Type] OR "interview"[Publication Type] OR "lectures"[Publication Type] OR "legal cases"[Publication Type] OR "legislation"[Publication Type] OR "letter"[Publication Type] OR "news"[Publication Type] OR "newspaper article"[Publication Type] OR "patient education handout"[Publication Type] OR "popular works"[Publication Type] OR "congresses"[Publication Type] OR "consensus development conference"[Publication Type] OR "consensus development conference, nih"[Publication Type] OR "practice guideline"[Publication Type]) NOT ("animals"[MeSH Terms] NOT "humans"[MeSH Terms]))**

|  | Concepts | Search Terms |
| --- | --- | --- |
| Concept Search | QOL OR HRQOL | ((((((((((("Quality of Life"[Mesh]) OR quality of life[tiab])) OR "health-related quality of life")) |
| Instrument Search | PRO OR Questionnaires OR Item Banks | (((((((("Patient Outcome Assessment"[Mesh]) OR ((patient-reported outcome*) OR (((patient-reported outcome) OR patient-reported outcome measure) OR patient-reported outcome assessment)))) OR patient-administ*) OR self-administ*) OR self-complet*)) OR (((("Questionnaires"[Mesh]) OR questionnaire*) OR item*) OR item bank*))) |
| Population Search | Singapore AND Adult | ((((Singapore [MeSH]) OR Singapore)) AND Adult [MeSH]))) |
| Precise filter | Terwee Precise filter for studies on measurement properties of measurement instruments | Search instrumentation[sh] OR Validation Studies[pt] OR "reproducibility of results"[MeSH Terms] OR reproducib*[tiab] OR "psychometrics"[MeSH] OR psychometr*[tiab] OR clinimetr*[tiab] OR clinometr*[tiab] OR "observer variation"[MeSH] OR observer variation[tiab] OR "discriminant analysis"[MeSH] OR reliab*[tiab] OR valid*[tiab] OR coefficient[tiab] OR "internal consistency"[tiab] OR (cronbach*[tiab] AND (alpha[tiab] OR alphas[tiab])) OR "item correlation"[tiab] OR "item correlations"[tiab] OR "item selection"[tiab] OR "item selections"[tiab] OR "item reduction"[tiab] OR "item reductions"[tiab] OR agreement[tw] OR precision[tw] OR imprecision[tw] OR "precise values"[tw] OR test-retest[tiab] OR (test[tiab] AND retest[tiab]) OR (reliab*[tiab] AND (test[tiab] OR retest[tiab])) OR stability[tiab] OR interrater[tiab] OR inter-rater[tiab] OR intrarater[tiab] OR intra-rater[tiab] OR intertester[tiab] OR inter-tester[tiab] OR intratester[tiab] OR intra-tester[tiab] OR interobserver[tiab] OR inter-observer[tiab] OR intraobserver[tiab] OR intra-observer[tiab] OR intertechnician[tiab] OR inter-technician[tiab] OR intratechnician[tiab] OR intra-technician[tiab] OR interexaminer[tiab] OR inter-examiner[tiab] OR intraexaminer[tiab] OR intra-examiner[tiab] OR interassay[tiab] OR inter-assay[tiab] OR intraassay[tiab] OR intra-assay[tiab] OR interindividual[tiab] OR inter-individual[tiab] OR intraindividual[tiab] OR intra-individual[tiab] OR interparticipant[tiab] OR inter-participant[tiab] OR intraparticipant[tiab] OR intra-participant[tiab] OR kappa[tiab] OR kappa's[tiab] OR kappas[tiab] OR "coefficient of variation"[tiab] OR repeatab*[tw] OR ((replicab*[tw] OR repeated[tw]) AND (measure[tw] OR measures[tw] OR findings[tw] OR result[tw] OR results[tw] OR test[tw] OR tests[tw])) OR generaliza*[tiab] OR generalisa*[tiab] OR concordance[tiab] OR (intraclass[tiab] AND correlation*[tiab]) OR discriminative[tiab] OR "known group"[tiab] OR "factor analysis"[tiab] OR "factor analyses"[tiab] OR "factor structure"[tiab] OR "factor structures"[tiab] OR dimensionality[tiab] OR subscale*[tiab] OR "multitrait scaling analysis"[tiab] OR "multitrait scaling analyses"[tiab] OR "item discriminant"[tiab] OR "interscale correlation"[tiab] OR "interscale correlations"[tiab] OR ((error[tiab] OR errors[tiab]) AND (measure*[tiab] OR correlat*[tiab] OR evaluat*[tiab] OR accuracy[tiab] OR accurate[tiab] OR precision[tiab] OR mean[tiab])) OR "individual variability"[tiab] OR "interval variability"[tiab] OR "rate variability"[tiab] OR "variability analysis"[tiab] OR (uncertainty[tiab] AND (measurement[tiab] OR measuring[tiab])) OR "standard error of measurement"[tiab] OR sensitiv*[tiab] OR responsive*[tiab] OR (limit[tiab] AND detection[tiab]) OR "minimal detectable concentration"[tiab] OR interpretab*[tiab] OR (small*[tiab] AND (real[tiab] OR detectable[tiab]) AND (change[tiab] OR difference[tiab])) OR "meaningful change"[tiab] OR "minimal important change"[tiab] OR "minimal important difference"[tiab] OR "minimally important change"[tiab] OR "minimally important difference"[tiab] OR "minimal detectable change"[tiab] OR "minimal detectable difference"[tiab] OR "minimally detectable change"[tiab] OR "minimally detectable difference"[tiab] OR "minimal real change"[tiab] OR "minimal real difference"[tiab] OR "minimally real change"[tiab] OR "minimally real difference"[tiab] OR "ceiling effect"[tiab] OR "floor effect"[tiab] OR "Item response model"[tiab] OR IRT[tiab] OR Rasch[tiab] OR "Differential item functioning"[tiab] OR DIF[tiab] OR "computer adaptive testing"[tiab] OR "item bank"[tiab] OR "cross-cultural equivalence"[tiab] |
| Exclusion Filter | Terwee Exclusion filter for studies on measurement properties of measurement instruments | (("addresses"[Publication Type] OR "biography"[Publication Type] OR "case reports"[Publication Type] OR "comment"[Publication Type] OR "directory"[Publication Type] OR "editorial"[Publication Type] OR "festschrift"[Publication Type] OR "interview"[Publication Type] OR "lectures"[Publication Type] OR "legal cases"[Publication Type] OR "legislation"[Publication Type] OR "letter"[Publication Type] OR "news"[Publication Type] OR "newspaper article"[Publication Type] OR "patient education handout"[Publication Type] OR "popular works"[Publication Type] OR "congresses"[Publication Type] OR "consensus development conference"[Publication Type] OR "consensus development conference, nih"[Publication Type] OR "practice guideline"[Publication Type]) NOT ("animals"[MeSH Terms] NOT "humans"[MeSH Terms])) |
