# Supplementary material for: Developing item banks to measure three important domains of health-related quality of life (HRQOL) in Singapore
Source: Health Qual Life Outcomes. 2020 Jan 2;18:2. doi: 10.1186/s12955-019-1255-1 (PMC6941315; doi:10.1186/s12955-019-1255-1)
Supplement: Supplementary file 3 — Additional file 3. Patient Reported Outcomes Measurement Instrument System (PROMIS) Domain Instruments included in the item library. [file 12955_2019_1255_MOESM3_ESM.docx]

# Additional file 3. Patient Reported Outcome Measurement Instrument System (PROMIS) Domain Instruments included in the item library

PROMIS Item Bank v1.0 – Emotional Distress – Anxiety

PROMIS Item Bank v1.0 – Emotional Distress – Depression

PROMIS Bank v1.0 – Psychosocial Illness Impact – Positive

PROMIS Bank v1.0 – Psychosocial Illness Impact – Negative

PROMIS Item Bank v1.0 – Self-Efficacy for Managing Emotions

PROMIS Item Bank v1.0 – Self-Efficacy for Managing Social Interactions

PROMIS Item Bank v1.0 – Self-Efficacy for Managing Daily Activities

PROMIS Bank v1.2 – Physical Function

PROMIS Bank v1.0 – Physical Function for Samples with Mobility Aid Users

PROMIS Item Bank v1.0 – Satisfaction with Participation in Discretionary Social Activities

PROMIS Item Bank v1.0 – Satisfaction with Participation in Social Roles

PROMIS Item Bank v2.0 – Satisfaction with Social Roles and Activities

PROMIS Item Bank v2.0 – Ability to Participate in Social Roles and Activities

PROMIS Item Bank v2.0 – Companionship

PROMIS Item Bank v2.0 – Informational Support

PROMIS Item Bank v2.0 – Emotional Support

PROMIS Item Bank v2.0 – Instrumental Support

PROMIS Item Bank v2.0 – Social Isolation
