# Supplementary material for: Developing item banks to measure three important domains of health-related quality of life (HRQOL) in Singapore
Source: Health Qual Life Outcomes. 2020 Jan 2;18:2. doi: 10.1186/s12955-019-1255-1 (PMC6941315; doi:10.1186/s12955-019-1255-1)
Supplement: Supplementary file 4 — Additional file 4. Instruments excluded from the item library. [file 12955_2019_1255_MOESM4_ESM.docx]

# Additional file 4. Instruments excluded from the item library

1. Generic Instruments

Instrumental Self Maintenance Scale

Katz Index

Knee Society Clinical Rating System

Questionnaire to measure transportation, occupation, leisure time, and household physical activities

Revised Memory and Behavioral Problems Checklist

Social Support Questionnaire

Visual Function Scale (14 Items)

Zarit Burden Interview

1. Disease-Specific Instruments

Asthma Control Questionnaire

Audit of Diabetes-Dependent Quality of Life

Eastern Cooperative Oncology Group Performance Status scale

Functional Assessment of Cancer Therapy - Cognitive Function

Hearing Handicap Inventory for the Elderly Screening Version questionnaire

Karnofsky Performance Status

Physical Activity Scale - Alzheimer’s Disease

Rhinoconjunctivitis Quality of Life Questionnaire

Standardized Asthma Quality of Life Questionnaire

Systemic Lupus Erythematosus Quality of Life Questionnaire

Tinnitus Handicap Inventory
