# Supplementary material for: Developing item banks to measure three important domains of health-related quality of life (HRQOL) in Singapore
Source: Health Qual Life Outcomes. 2020 Jan 2;18:2. doi: 10.1186/s12955-019-1255-1 (PMC6941315; doi:10.1186/s12955-019-1255-1)
Supplement: Supplementary file 5 — Additional file 5. Domain-specific item banks. [file 12955_2019_1255_MOESM5_ESM.docx]

# Additional file 5. Domain-specific item banks

1. Physical Functioning (© copyright Singapore General Hospital)

| Item No. | Item |
| --- | --- |
| 1 | I am able to feed myself without assistance from other people |
| 2 | I am able to wash my entire body without assistance from other people |
| 3 | I am able to bathe myself without assistance from other people |
| 4 | I am able to towel-dry my entire body after bathing, without assistance from other people |
| 5 | I am able to dress without limiting myself to clothes that are easy to put on, without assistance from other people |
| 6 | I am able to put on a shirt or a blouse without assistance from other people |
| 7 | I am able to put on pants without assistance from other people |
| 8 | I am able to put on and take off my socks without assistance from other people |
| 9 | I am able to tie my shoelaces without assistance from other people |
| 10 | I am able to comb my hair, shave my face, or put on make-up without assistance from other people |
| 11 | I am able to use my hands and fingers |
| 12 | I am able to button my shirt or blouse without assistance from other people |
| 13 | I am able to hold a pen to write |
| 14 | I am able to use a spoon or a fork |
| 15 | I am able to use a pair of chopsticks |
| 16 | I am able to use a nail clipper to trim my fingernails |
| 17 | I am able to open a tight jar lid |
| 18 | I am able to pick up coins from a table top |
| 19 | I am able to hold a glass full of water without spilling it, while staying still |
| 20 | I am able to stand, unsupported, on tiptoes |
| 21 | I am able to kneel on the floor |
| 22 | I am able to squat and get up without assistance from other people |
| 23 | I am able to jump up and down |
| 24 | I am able to bend down (e.g. when I pick up items from the floor) |
| 25 | I am able to roll onto my stomach, while lying in bed, without assistance from other people |
| 26 | I am able to get in and out of a car without assistance from other people |
| 27 | I am able to get in and out of bed without assistance from other people |
| 28 | I am able to get in and out of a chair without assistance from other people |
| 29 | I am able to get on and off a toilet seat without assistance from other people |
| 30 | I am able to use the toilet bowl without assistance from other people |
| 31 | I am able to stand up from sitting on an armless chair without assistance from other people |
| 32 | I am able to **climb up the stairs** of an overhead bridge without assistance from other people |
| 33 | I am able to **go down the stairs** of an overhead bridge without assistance from other people |
| 34 | I am able to move around at home without assistance from other people |
| 35 | I am able to move around **in my neighbourhood** without assistance from other people |
| 36 | I am able to move around **beyond my neighbourhood** without assistance from other people |
| 37 | I am able to walk on a slippery floor without assistance from other people (e.g. in the toilet or wet market) |
| 38 | I am able to run 1 round on the running track (approximately 400 metres) |
| 39 | I am able to run a short distance (e.g.to catch a bus) |
| 40 | I am able to exercise for 20 minutes |
| 41 | I am able to run a half marathon (21 kilometres) |
| 42 | I am able to run a full marathon (42 kilometres) |
| 43 | I am able to take things out of a high cupboard without assistance from other people |
| 44 | I am able to lift a full glass of water to my mouth |
| 45 | I am able to carry 2 bags filled with groceries |
| 46 | I am able to **pull open** a heavy door without assistance from other people |
| 47 | I am able to **push open** a heavy door without assistance from other people |
| 48 | I am able to do low intensity physical activities (e.g. strolling around my neighbourhood or watering a house plant) |
| 49 | I am able to do moderate intensity physical activities (e.g. brisk walking or cycling on a stationary bicycle) |
| 50 | I am able to do high intensity physical activities (e.g. fast cycling, running, or sprinting) |
| 51 | I am able to do vigorous physical activities (e.g. full triathlons/marathons or dragon boat racing) |
| 52 | I am able to prepare a simple meal without assistance from other people |
| 53 | I am able to wash dishes by hand |
| 54 | I am able to mop, vacuum, or sweep floors |
| 55 | I am able to scrub floors with a brush (e.g. scrubbing the toilet floor) |
| 56 | I am able to do laundry |
| 57 | I am able to water a house plant |
| 58 | I am able move about on my own without assistance from other people |
| 59 | I am able to move around without a walking aid (e.g. walking stick, walking frame, umbrella) |
| 60 | I am able to stand unsupported for 15 minutes (e.g. while waiting in a line, waiting for a bus) |
| 61 | I am bedridden |

1. Social Relationships (© copyright Singapore General Hospital)

| Item No. | Item |
| --- | --- |
| 1 | I have a good relationship with my family |
| 2 | I participate in family activities |
| 3 | I have gatherings with my family |
| 4 | I feel loved and cared for by my family |
| 5 | Overall, my family supports me when I need it |
| 6 | My family is willing to listen when I need to talk about my worries and problems |
| 7 | My family is willing to help with my daily tasks (e.g. shopping, giving me a ride, or helping me with household tasks) when I need it |
| 8 | My family is willing to give me information when I need it |
| 9 | I give support to my family |
| 10 | I take care of my family |
| 11 | I communicate well with my family |
| 12 | I keep in touch with my family |
| 13 | I spend time with my family |
| 14 | Overall, my friends support me when I need it |
| 15 | I give support to my friends |
| 16 | I keep in touch with my friends |
| 17 | I communicate well with my friends |
| 18 | I have gatherings with my circle of friends |
| 19 | People turn up when I organize social activities and gatherings |
| 20 | I spend time with my friends |
| 21 | Overall, my spouse/partner supports me when I need it |
| 22 | I rely on my spouse/partner when I need help |
| 23 | I give support to my spouse/partner |
| 24 | My spouse/partner relies on me when he/she needs help |
| 25 | My spouse/partner and I communicate openly with each other |
| 26 | My spouse/partner and I are able to freely talk about our feelings |
| 27 | I have someone to do my chores (e.g. grocery shopping) if I were unable to do it myself |
| 28 | I have someone to run my errands if I were unable to do it myself |
| 29 | I have someone to buy or cook food for me if I were unable to do it myself |
| 30 | I have someone to help me with my personal care if I were unable to do it myself (e.g. feeding, bathing, dressing, grooming, or toileting) |
| 31 | I have someone I can go to for advice if I need it |
| 32 | I have someone to talk to about my problems |
| 33 | I have someone who can provide me with information if I need it |
| 34 | I know that I have someone to help me if I have financial difficulties |
| 35 | I give help to others |
| 36 | I take care of others |
| 37 | I get along well with others |
| 38 | I manage conflict with others well |
| 39 | I live harmoniously with others |
| 40 | I communicate well with others |
| 41 | I keep in touch with others |
| 42 | I have a social life |
| 43 | I am able to socialize with others |
| 44 | I have someone who would spend time with me when I need company |
| 45 | I feel loved and cared for |
| 46 | Overall, I am happy with my family relationships |
| 47 | Overall, I am satisfied with the support I get from my family |
| 48 | Overall, I am satisfied with the support I get from my friends |
| 49 | Overall, I am satisfied with the support I get from my spouse/partner |
| 50 | Overall, I am satisfied with the support I give to others |
| 51 | Overall, I am satisfied with how well I communicate with others |

1. Positive Mindset (© copyright Singapore General Hospital)

| Item No. | Item |
| --- | --- |
| 1 | I am able to accept people as they are |
| 2 | I am able to accept the way things work out |
| 3 | I am able to accept who I am |
| 4 | I am able to solve my own problems |
| 5 | I am able to appreciate the people in my life |
| 6 | I am able to appreciate each day fully |
| 7 | I am able to appreciate what each day brings |
| 8 | I feel calm |
| 9 | I am a cheerful person |
| 10 | I don’t give up when things look hopeless |
| 11 | I am able to bounce back after setbacks |
| 12 | I am able to cope with life's challenges |
| 13 | I am able to deal with stress |
| 14 | Overall, I feel contented with my life |
| 15 | Overall, I am satisfied with my life |
| 16 | I am able to see the good in everyone |
| 17 | I am able to handle my negative feelings |
| 18 | I am able to manage my anger |
| 19 | I do not let my worries overwhelm me |
| 20 | I am able to manage my worries |
| 21 | I feel in control of my life |
| 22 | I feel enthusiastic |
| 23 | I feel enthusiastic about life |
| 24 | I enjoy life |
| 25 | I find comfort in my religion or spiritual beliefs |
| 26 | I find comfort in my religious beliefs |
| 27 | Overall, I feel happy |
| 28 | I try to see the funny side of stressful situations |
| 29 | I try to see the lighter side of stressful situations |
| 30 | I am able to see things positively |
| 31 | I am able to relax |
| 32 | Overall, I feel confident |
| 33 | I feel hopeful about the future |
| 34 | I am able to motivate myself |
| 35 | I worry about not being accepted by others |
| 36 | I am easily discouraged |
| 37 | I am bothered by small matters |
| 38 | I tend to overthink things |
| 39 | I give up easily |
| 40 | I lose my temper easily |
| 41 | In most ways my life is close to my ideal |
| 42 | I am clear about what I want in life |
| 43 | I feel at peace with myself |
| 44 | I feel that my life has a purpose |
| 45 | I live a meaningful life |
| 46 | I take pride in my achievements |
| 47 | I believe I am a good person |
| 48 | I am not a bad person |
