# Supplementary material for: Developing item banks to measure three important domains of health-related quality of life (HRQOL) in Singapore
Source: Health Qual Life Outcomes. 2020 Jan 2;18:2. doi: 10.1186/s12955-019-1255-1 (PMC6941315; doi:10.1186/s12955-019-1255-1)
Supplement: Supplementary file 6 — Additional file 6 Table S1. Means and standard deviations for all tested intermediate descriptors. [file 12955_2019_1255_MOESM6_ESM.docx]

# Additional file 6: Table S1. Means and standard deviations for all tested intermediate descriptors

Capability 1

| **Variable** | **Obs** | **Mean** | **Std. Dev.** | **Min** | **Max** | **Mode** |
| --- | --- | --- | --- | --- | --- | --- |
| With extreme difficulty | 30 | 8.10 | 14.43 | 0 | 76 | 0 |
| With major difficultly | 30 | 9.10 | 8.77 | 0 | 40 | 2 |
| With great difficulty | 30 | 9.57 | 12.76 | 0 | 72 | 7 |
| With a lot of difficulty | 30 | 10.27 | 8.76 | 0 | 38 | 5 |
| With very much difficulty | 30 | 10.53 | 15.99 | 1 | 89 | 6 |
| **With much difficulty** | **30** | **17.17** | **14.06** | **2** | **50** | **5** |
| With relative difficulty | 30 | 45.73 | 19.33 | 3 | 96 | 42 |
| With partial difficulty | 30 | 52.23 | 20.49 | 10 | 95 | 49 |
| **With moderate difficulty** | **30** | **52.83** | **16.59** | **14** | **83** | **55** |
| With fair difficulty | 30 | 53.07 | 19.72 | 17 | 96 | 45 |
| With some difficulty | 30 | 59.63 | 19.70 | 24 | 94 | 45 |
| With mild difficulty | 30 | 65.30 | 25.77 | 8 | 100 | 69 |
| With slight difficulty | 30 | 77.20 | 24.04 | 13 | 99 | 96 |
| **With minor difficulty** | **30** | **80.03** | **18.88** | **3** | **98** | **79** |
| With a little difficulty | 30 | 80.30 | 21.52 | 6 | 99 | 93 |
| With very little difficulty | 30 | 81.13 | 24.21 | 2 | 99 | 90 |
| With a bit of difficulty | 30 | 81.17 | 19.74 | 23 | 100 | 89 |
| With not much difficulty | 30 | 85.60 | 12.64 | 53 | 100 | 90 |

Capability 2

| **Variable** | **Obs** | **Mean** | **Std. Dev.** | **Min** | **Max** | **Mode** |
| --- | --- | --- | --- | --- | --- | --- |
| Little ability to do | 26 | 18.23 | 19.55 | 1 | 76 | 4 |
| Minimally able to do | 30 | 19.53 | 17.01 | 2 | 64 | 2 |
| Limited ability to do | 30 | 25.70 | 21.05 | 1 | 98 | 16 |
| **Slightly able to do** | **30** | **27.00** | **19.83** | **2** | **68** | **25** |
| Mildly able to do | 30 | 31.07 | 21.20 | 2 | 81 | 19 |
| Partially able to do | 30 | 41.60 | 15.70 | 10 | 70 | 44 |
| Fairly able to do | 30 | 46.30 | 17.19 | 3 | 80 | 50 |
| **Moderately able to do** | **30** | **51.20** | **10.62** | **33** | **81** | **47** |
| Substantially able to do | 30 | 63.27 | 22.06 | 17 | 97 | 59 |
| Able to do | 30 | 84.00 | 19.70 | 41 | 100 | 100 |
| Mostly able to do | 30 | 85.43 | 10.06 | 62 | 100 | 95 |
| Largely able to do | 26 | 89.69 | 9.23 | 67 | 100 | 100 |
| Greatly able to do | 30 | 91.83 | 7.08 | 78 | 100 | 98 |
| Fully able to do | 30 | 94.87 | 17.06 | 7 | 100 | 100 |

Frequency

| **Variable** | **Obs** | **Mean** | **Std. Dev.** | **Min** | **Max** | **Mode** |
| --- | --- | --- | --- | --- | --- | --- |
| Almost never | 30 | 7.13 | 9.94 | 0 | 44 | 1 |
| Hardly | 30 | 14.63 | 17.64 | 0 | 87 | 14 |
| Rarely | 30 | 15.93 | 17.03 | 0 | 60 | 3 |
| **Seldom** | **30** | **20.37** | **11.48** | **3** | **45** | **17** |
| Infrequently | 30 | 24.17 | 18.23 | 2 | 67 | 8 |
| Once in a while | 30 | 28.30 | 17.31 | 2 | 64 | 35 |
| At times | 30 | 33.87 | 18.37 | 2 | 72 | 31 |
| Occasionally | 30 | 37.63 | 19.07 | 2 | 98 | 34 |
| Some of the time | 30 | 39.10 | 17.81 | 2 | 70 | 27 |
| **Sometimes** | **30** | **39.70** | **17.62** | **7** | **85** | **50** |
| Every now and then | 30 | 59.97 | 20.05 | 25 | 100 | 51 |
| Many a time | 30 | 75.87 | 15.26 | 38 | 100 | 71 |
| **Usually** | **30** | **77.67** | **14.32** | **44** | **100** | **84** |
| Often | 30 | 81.07 | 18.35 | 25 | 100 | 87 |
| A lot of the time | 30 | 81.87 | 12.38 | 55 | 99 | 71 |
| Regularly | 30 | 82.43 | 16.69 | 27 | 100 | 100 |
| Frequently | 30 | 84.30 | 16.34 | 38 | 100 | 100 |
| Most of the time | 30 | 84.70 | 16.23 | 19 | 100 | 96 |

Intensity

| **Variable** | **Obs** | **Mean** | **Std. Dev.** | **Min** | **Max** | **Mode** |
| --- | --- | --- | --- | --- | --- | --- |
| Very little | 26 | 5.96 | 4.49 | 0 | 19 | 3 |
| A little | 30 | 11.37 | 9.69 | 1 | 39 | 11 |
| Hardly | 30 | 14.00 | 18.99 | 0 | 96 | 19 |
| A little bit | 30 | 16.90 | 21.72 | 0 | 97 | 2 |
| Not much | 30 | 18.30 | 16.48 | 0 | 67 | 22 |
| Slightly | 30 | 22.40 | 19.18 | 2 | 76 | 2 |
| **Mildly** | **30** | **27.83** | **18.86** | **2** | **81** | **46** |
| So so | 30 | 34.30 | 17.00 | 0 | 56 | 48 |
| Somewhat | 30 | 36.57 | 17.39 | 3 | 61 | 52 |
| Partially | 30 | 37.83 | 13.56 | 11 | 56 | 50 |
| Fairly | 30 | 40.60 | 18.89 | 2 | 72 | 50 |
| Quite a bit | 30 | 44.27 | 25.70 | 2 | 88 | 71 |
| More or less | 30 | 46.13 | 16.12 | 3 | 92 | 50 |
| **Moderately** | **30** | **46.67** | **10.49** | **22** | **71** | **51** |
| Mostly | 30 | 74.20 | 22.46 | 7 | 100 | 83 |
| **Quite a lot** | **30** | **78.40** | **15.27** | **23** | **99** | **73** |
| Very much | 30 | 83.17 | 15.69 | 33 | 100 | 89 |
| Largely | 30 | 85.40 | 12.83 | 52 | 100 | 96 |
| Greatly | 30 | 87.13 | 11.57 | 62 | 100 | 100 |
