# Supplementary material for: Developing item banks to measure three important domains of health-related quality of life (HRQOL) in Singapore
Source: Health Qual Life Outcomes. 2020 Jan 2;18:2. doi: 10.1186/s12955-019-1255-1 (PMC6941315; doi:10.1186/s12955-019-1255-1)
Supplement: Supplementary file 7 — Additional file 7 TableS2. Items for which instrument developer permission was sought. [file 12955_2019_1255_MOESM7_ESM.docx]

# Additional file 7: Table S2. Items for which instrument developer permission was sought

|  | Instrument Acronym | Item No. (Original Instrument) | Item (Original Instrument) | SHAWS Item No. | Adapted Item  (Item calibration) | Domain |
| --- | --- | --- | --- | --- | --- | --- |
| 1 | CES-D | 8 | I felt hopeful about the future | 33 | I feel hopeful about the future | Positive Mindset |
| 2 | CQOLC | 34 | I am satisfied with the support I get from my family. | 47 | Overall, I am satisfied with the support I get from my family | Social Relationships |
| 3 | HADS | 6 | I feel cheerful | 9 | I am a cheerful person | Positive Mindset |
| 4 | HADS | 4 | I can laugh and see the funny side of things | 28 | I try to see the funny side of stressful situations | Positive Mindset |
| 5 | EQ-5D-3L | M3 | I am confined to bed. | 61 | I am bedridden | Physical Functioning |
| 6 | PROMIS-APSRA | SRPPER43r1 | I have trouble keeping in touch with others | 41 | I keep in touch with others | Social Relationships |
| 7 | PROMIS-ES | FSE31059x2 | I have someone to confide in or talk to about myself or my problems | 32 | I have someone to talk to about my problems | Social Relationships |
| 8 | PROMIS-InfS | FSE31058x2 | I have someone to give me information if I need it | 33 | I have someone who can provide me with information if I need it | Social Relationships |
| 9 | PROMIS-InsS | SS6 | Do you have someone to run errands if you need it? | 28 | I have someone to run my errands if I were unable to do it myself | Social Relationships |
| 10 | PROMIS-Mobility Aid | PF_14 | Are you able to roll onto your stomach while lying in bed? | 25 | I am able to roll onto my stomach, while lying in bed, without assistance from other people | Physical Functioning |
| 11 | PROMIS-PF | PFA44 | Are you able to put on a shirt or blouse? | 6 | I am able to put on a shirt or a blouse without assistance from other people | Physical Functioning |
| 12 | PROMIS-PF | PFB17 | Are you able to put on and take off your socks? | 8 | I am able to put on and take off my socks without assistance from other people | Physical Functioning |
| 13 | PROMIS-PF | PFA43 | Are you able to write with a pen or pencil? | 13 | I am able to hold a pen to write | Physical Functioning |
| 14 | PROMIS-PF | PFB41 | Are you able to trim your fingernails? | 16 | I am able to use a nail clipper to trim my fingernails | Physical Functioning |
| 15 | PROMIS-PF | PFB21 | Are you able to pick up coins from a table top? | 18 | I am able to pick up coins from a table top | Physical Functioning |
| 16 | PROMIS-PF | PFB40 | Are you able to stand up on tiptoes? | 20 | I am able to stand, unsupported, on tiptoes | Physical Functioning |
| 17 | PROMIS-PF | PFC40 | Are you able to kneel on the floor? | 21 | I am able to kneel on the floor | Physical Functioning |
| 18 | PROMIS-PF | PFA41 | Are you able to squat and get up? | 22 | I am able to squat and get up without assistance from other people | Physical Functioning |
| 19 | PROMIS-PF | PFB9 | Are you able to jump up and down? | 23 | I am able to jump up and down | Physical Functioning |
| 20 | PROMIS-PF | PFA9 | Are you able to bend down and pick up clothing from the floor? | 24 | I am able to bend down (e.g. when I pick up items from the floor) | Physical Functioning |
| 21 | PROMIS-PF | PFA56 | Are you able to get in and out of a car? | 26 | I am able to get in and out of a car without assistance from other people | Physical Functioning |
| 22 | PROMIS-PF | PFB24 | Are you able to run a short distance, such as to catch a bus? | 39 | I am able to run a short distance (e.g. to catch a bus) | Physical Functioning |
| 23 | PROMIS-PF | PFA17 | Are you able to reach into a high cupboard? | 43 | I am able to take things out of a high cupboard without assistance from other people | Physical Functioning |
| 24 | PROMIS-PF | PFB29 | Are you able to lift a full cup or glass to your mouth? | 44 | I am able to lift a full glass of water to my mouth | Physical Functioning |
| 25 | PROMIS-PF | PFB8r1 | Are you able to carry two bags filled with groceries 100 yards (100 m)? | 45 | I am able to carry 2 bags filled with groceries | Physical Functioning |
| 26 | PROMIS-PF | PFA12 | Are you able to push open a heavy door? | 47 | I am able to **push open** a heavy door without assistance from other people | Physical Functioning |
| 27 | PROMIS-PF | PFC49 | Are you able to water a house plant? | 57 | I am able to water a house plant | Physical Functioning |
| 28 | PROMIS-PII-Positive | II25 | I am able to accept the way things work out | 2 | I am able to accept the way things work out | Positive Mindset |
| 29 | PROMIS-PII-Positive | II19 | I can appreciate people in my life | 5 | I am able to appreciate the people in my life | Positive Mindset |
| 30 | PROMIS-PII-Positive | II35 | I can appreciate each day fully | 6 | I am able to appreciate each day fully | Positive Mindset |
| 31 | PROMIS-PII-Positive | II29 | I am able to deal with stress and problems | 13 | I am able to deal with stress | Positive Mindset |
| 32 | PROMIS-PII-Positive | II34 | I am able to enjoy life | 24 | I enjoy life | Positive Mindset |
| 33 | PROMIS-PII-Positive | II39 | I have a sense of purpose in life | 44 | I feel that my life has a purpose | Positive Mindset |
| 34 | PROMIS-PII-Positive | II5 | I believe I am a good person | 44 | I believe I am a good person | Positive Mindset |
| 35 | PROMIS-SE-MDA | SEMDA002 | I can eat without help from anyone. | 1 | I am able to feed myself without assistance from other people | Physical Functioning |
| 36 | PROMIS-SE-MDA | SEMDA006 | I can get in and out of a chair. | 28 | I am able to get in and out of a chair without assistance from other people | Physical Functioning |
| 37 | PROMIS-SE-MDA | SEMDA012 | I can exercise at a moderate level for 10 minutes (for example: walking briskly, biking, swimming, aerobics). | 49 | I am able to do moderate intensity physical activities (e.g. brisk walking or cycling on a stationary bicycle) | Physical Functioning |
| 38 | PROMIS-SE-MDA | SEMDA009 | I can stand for 5 minutes (for example: waiting in a line, waiting for a bus). | 60 | I am able to stand unsupported for 15 minutes (e.g. while waiting in a line, waiting for a bus) | Physical Functioning |
| 39 | PROMIS-SE-MDA | SEMDA028 | I can take care of others (for example: cook for others, help them dress, watch children). | 36 | I take care of others | Social Relationships |
| 40 | PROMIS-SE-ME | SEMEM015 | I can handle negative feelings. | 17 | I am able to handle my negative feelings | Positive Mindset |
| 41 | PROMIS-SE-ME | SEMEM001 | I can keep anxiety from becoming overwhelming. | 20 | I am able to manage my worries | Positive Mindset |
| 42 | PROMIS-SE-MSI | SEMSS005 | I can keep in touch with friends and family. | 12 | I keep in touch with my family | Social Relationships |
| 43 | PROMIS-SE-MSI | SEMSS005 | I can keep in touch with friends and family. | 16 | I keep in touch with my friends | Social Relationships |
| 44 | PROMIS-SE-MSI | SEMSS010 | If I need help, I have someone to help with my financial affairs. | 34 | I know that I have someone to help me if I have financial difficulties | Social Relationships |
| 45 | SSAQ | XIV.OL14 | When things look hopeless, I don’t give up | 10 | I don’t give up when things look hopeless | Positive Mindset |
| 46 | SSAQ | XIV.OL27 | I take pride in my achievements | 46 | I take pride in my achievements | Positive Mindset |
| 47 | SSAQ | VI.Soc5.c | Do your spouse, children, close friends and/or relatives help with daily tasks like shopping, giving you a ride, or helping you with household tasks? | 7 | My family is willing to help with my daily tasks (e.g. shopping, giving me a ride, or helping me with household tasks) when I need it | Social Relationships |
| 48 | SSAQ | VI.Soc5.b | Are your spouse, children, close friends and/or relatives willing to listen when you need to talk about your worries or problems? | 6 | My family is willing to listen when I need to talk about my worries and problems | Social Relationships |
| 49 | SHAQ | 3a | Are you able to: Use a spoon or fork? | 14 | I am able to use a spoon or a fork | Physical Functioning |
| 50 | SHAQ | 3c | Are you able to: Use a pair of chopsticks? | 15 | I am able to use a pair of chopsticks | Physical Functioning |
| 51 | SHAQ | 5c | Are you able to: Get on and off the toilet seat? | 29 | I am able to get on and off a toilet seat without assistance from other people | Physical Functioning |
| 52 | STAI | 1 | I feel calm | 8 | I feel calm | Positive Mindset |
| 53 | SWLS | 1 | In most ways my life is close to my ideal | 41 | In most ways my life is close to my ideal | Positive Mindset |

**CES-D:** Center for Epidemiologic Studies Depression Scale

**CQOLC:** Caregiver Quality of Life Index-Cancer

**EQ-5D-3L:** EuroQoL 5 Dimension 3 Level Instrument

**HADS:** Hospital Anxiety and Depression Scale

**PROMIS-APSRA:** PROMIS Item Bank v2.0 – Ability to Participate in Social Roles and Activities

**PROMIS-ES:** PROMIS Item Bank v2.0 – Emotional Support

**PROMIS-InfS:** PROMIS Item Bank v2.0 – Informational Support

**PROMIS-InsS**: PROMIS Item Bank v2.0 – Instrumental Support

**PROMIS-Mobility Aid:** PROMIS Bank v1.0 – Physical Function for Samples with Mobility Aid Users

**PROMIS-PF:** PROMIS Bank v1.2 – Physical Function

**PROMIS-PII-Positive:** PROMIS Bank v1.0 – Psychosocial Illness Impact – Positive

**PROMIS-SE-MDA:** PROMIS Item Bank v1.0 – Self-Efficacy for Managing Daily Activities

**PROMIS-SE-ME:** PROMIS Item Bank v1.0 – Self-Efficacy for Managing Emotions

**PROMIS-SE-MSI:** PROMIS Item Bank v1.0 – Self-Efficacy for Managing Social Interactions

**SHAQ:** Scleroderma Health Assessment Questionnaire

**SSAQ:** Singapore Successful Aging Questionnaire

**STAI:** State-Trait Anxiety Inventory

**SWLS:** Satisfaction with Life Scale
